# Driving restrictions for Dutch patients with an implantable cardioverter defibrillator: Compliance and associated factors

**DOI:** 10.1007/s12471-017-1067-z

**Published:** 2017-12-19

**Authors:** N. Jongejan, I. Timmermans, J. Elders, K. Meijer, M. Meine, P. A. Doevendans, H. Versteeg, A. E. Tuinenburg

**Affiliations:** 1 Department of Cardiology, University Medical Center Utrecht, Utrecht, The Netherlands; 2 CoRPS—Center of Research on Psychology in Somatic Diseases, Department of Medical Psychology, Tilburg University, Tilburg, The Netherlands; 3 Department of Cardiology, Canisius Wilhelmina Hospital, Nijmegen, The Netherlands; 4 Department of Cardiology, Academic Medical Center Amsterdam, Amsterdam, The Netherlands; 5 Netherlands Heart Institute, Utrecht, The Netherlands

**Keywords:** Implantable cardioverter defibrillator, Driving restrictions, Compliance

## Abstract

**Background:**

Dutch patients with an implantable cardioverter defibrillator (ICD) are restricted from driving for two months after implantation or shocks. This requires significant lifestyle adjustments and is one of the primary concerns of ICD patients. Previous studies indicated that compliance with the driving restrictions is poor, but insight in socio-demographic, clinical and psychological factors associated with compliance is limited. Hence, this study aimed to explore compliance with the driving restrictions and associated factors in a large sample of Dutch ICD patients.

**Method:**

Dutch ICD patients (*N* = 313) completed an elaborative set of questionnaires at time of implantation and at four months after implantation, assessing socio-demographic, psychological and driving-related characteristics. Clinical data were collected from the patients’ medical records.

**Results:**

A substantial subgroup (28%) of the patient sample (median age 64 (interquartile range = 55–71), 81% male) reported to have been noncompliant with the driving restrictions. Univariate analysis indicated that noncompliant patients more often considered refusing the ICD due to the restrictions, compared to compliant patients (19% versus 10%, *p* = 0.02). Multivariate analysis showed that the feeling of understanding the reason behind the driving restrictions was associated with better compliance (odds ratio = 2.16, 95% confidence interval 1.02–4.56, *p* = 0.04). No other socio-demographic, clinical, psychological or driving-related factors were associated with compliance.

**Conclusion:**

A large number of ICD patients does not comply with the driving restrictions after implantation. This study emphasised the importance of the patient’s feeling of understanding the reason behind the restrictions.

## Introduction

Implantable cardiac defibrillator (ICD) therapy is the first-line treatment in the prevention of sudden cardiac death caused by life-threatening ventricular arrhythmias [1]. Despite its medical benefits the ICD imposes some restrictions on patients that may influence their daily lives. For example, patients with an ICD are restricted from driving a motor vehicle for a set period after the implantation and after ICD shocks, as they have an ongoing risk of sudden incapacitation that might harm others and themselves when driving [2]. Importantly, this risk is mainly a consequence of the underlying disease and not of the presence of an ICD [2].

The driving restrictions require significant lifestyle adjustments and are typically one of the primary concerns of ICD patients and their families [3]. In quantitative studies, patients have reported decreased self-esteem, relationship problems, a sense of loss of independence and social isolation due to the driving restrictions [4, 5]. This might explain why compliance with these restrictions is poor among ICD patients [6–9].

Compliance with driving restrictions and its associated factors have hardly been studied in European samples, and previous research in this field was conducted more than a decade ago [6–9]. Better insight into the factors related to driving after ICD implantation might improve individualised and structured information provision and support for patients, which could eventually lead to better compliance. Hence, the aim of this large quantitative study is to examine: 1) compliance rates; and 2) socio-demographic, clinical and psychological factors associated with compliance with driving restrictions in Dutch patients with an ICD.

## Method

### Dutch driving restrictions after ICD implantation

Dutch driving restrictions were first published in 2000 [10]. Patients with a driving license for motor vehicles >3,500 kg are permanently prohibited from driving, as well as patients who transport passengers in a professional setting. Patients with a driving license for motor vehicles <3,500 kg are restricted from driving for two months after implantation. Also, passenger transport (in volunteer settings) is limited to a maximum of nine passengers and only on special request; professional driving is allowed for <4 h/day. Patients receive a suitability statement from their cardiologist if their ICD did not deliver a shock and if no severe haemodynamic problems occurred during anti-tachycardia pacing in the first two months after implantation. In addition, no severe heart failure symptoms are allowed. With this statement, patients can obtain a special driving license (i. e., ‘code 100’ for private driving and ‘code 101’ for professional driving) from the Dutch driving licensing centre (*Centraal Bureau Rijvaardigheidsbewijzen*—CBR). This procedure takes another few weeks. Code 100 and code 101 licenses are valid for five years, instead of the standard ten for a regular driving license. If the ICD delivers one or more appropriate or inappropriate shocks, the actual restriction of these driving licenses implies that a patient is unfit to drive for at least two months after the last shock.

### Study design and participants

In this prospective multicentre cohort study in three Dutch Hospitals (University Medical Center Utrecht, Canisius Wilhelmina Hospital Nijmegen and Academic Medical Center Amsterdam), consecutive patients receiving a first-time ICD with or without cardioverter resynchronisation therapy (CRT) were included between December 2012 and March 2015. Patients with a valid driving license were eligible for participation. We excluded patients on a waiting list for heart transplantation, patients with cognitive impairment, patients with insufficient knowledge of the Dutch language to complete questionnaires and patients with a life expectancy of <1 year. Approval of local institutional review boards was obtained and the study was conducted in accordance with the Declaration of Helsinki (Brazil, October 2013). All patients provided written informed consent. Patients received a baseline questionnaire one day after implantation, and were asked to return it within two weeks. Four months after implantation all patients received a follow-up questionnaire.

## Measures

### Socio-demographic and clinical variables

Information on socio-demographic variables was obtained from purpose-designed questions in the baseline questionnaire. Clinical information was collected from the patients’ medical records.

### Compliance with driving restrictions

Compliance was measured by the following purpose-designed questions in the follow-up questionnaire: ‘Have you driven a motor vehicle before receiving a code 100 driving license?’ and ‘How many weeks after implantation did you resume driving a motor vehicle?’ If a patient answered the first question with a ‘yes’ and/or the second question with ‘< eight weeks’, he was classified as noncompliant.

### Driving behaviour before ICD implantation

Patients’ driving behaviour before ICD implantation was assessed using purpose-designed questions in the baseline questionnaires. Patients were asked which driving license(s) they have, if their partner has a driving license, if they are the main driver in their family, how many days per week they drive a motor vehicle, how many kilometres per week they drive, if driving is mostly for work or private purposes, and if they mostly drive within or outside urbanised areas.

### Information provision regarding the driving restrictions

Information provision about the driving restrictions was measured with purpose-designed questions in the follow-up questionnaire. Patients were asked whether they received information about the restrictions, in what way, from whom and at which moment. They were asked if the information provision was sufficient, if the reason behind the driving restriction was clear to them, whether they felt the driving restrictions were acceptable, and if they had considered refusing ICD implantation because of the restrictions.

### Psychological variables

The* distressed (Type D) personality*, a combined tendency towards negative affectivity and social inhibition, was assessed with the 14-item Type D Scale (DS14). The items on this scale are rated on a 5-point Likert scale ranging from 0 (false) to 4 (true) and can be divided into two subscales: negative affectivity and social inhibition [11]. A standardised cut-off score of ≥10 on both subscales was used to classify patients with Type D [11].

*Anxiety symptoms* were measured using the 7‑item Generalised Anxiety Disorder (GAD-7) scale. Items on this scale are rated on a 4-point Likert scale ranging from 0 (not at all) to 3 (almost daily) [12]. A cut-off value of ≥10 was used to identify patients with anxiety [12].

The 9‑item Patient Health Questionnaire (PHQ-9) was used to assess *depressive symptoms*. This questionnaire scores each of the nine DSM-IV criteria for depression on a 4-point Likert scale from 0 (not at all) to 3 (nearly every day) [13]. A cut-off score of ≥10 was used to classify patients with depression [13].

*ICD concerns* were measured using the 8‑item ICD concerns questionnaire (ICDC) [14, 15]. Items are scored on a 5-point Likert scale from 0 (not at all) to 4 (very much so). The scale yields a score for severity of concerns (0–32). A higher score indicates more severe concerns [14, 15].

*Loneliness* was measured using the 10-item University of California, Los Angeles Loneliness Scale (UCLA-R-S). This scale consists of 20 items rated on a 4-point Likert scale ranging from 1 (never) to 4 (very often). The higher the patient’s score, the more loneliness he or she experiences [16].

## Statistical analyses

Baseline characteristics of compliant and noncompliant patients were compared using Fisher’s exact tests for discrete variables and Mann-Whitney U tests for continuous variables. Frequencies with percentages (*N* (%)) are reported for categorical variables and medians with interquartile ranges (median (interquartile range—IQR)) for continuous variables. Multiple logistic regression analysis was used to evaluate the association between patient characteristics and compliance with driving restrictions. The level of statistical significance was set at *p* < 0.05, and all analyses were performed with SPSS 22.0 for Windows (SPSS Inc., Chicago, Illinois).

## Results

### Patient characteristics and compliance rate

The sample (*N* = 313) had a median age of 64 (IQR = 55–71) years, and the majority was male (81%). Most patients received an ICD for primary prevention (69%). Median left ventricular ejection fraction was 29% (IQR = 23–35) and 47% of the patients were classified as having mild heart failure symptoms (New York Heart Association (NYHA) class II) at baseline. Prevalence of depression and anxiety was 22 and 15%, respectively, and 14% of the sample was classified as having a Type D personality. Median time between receiving the suitability statement from their cardiologist and receiving the special driving license was 2 weeks (IQR = 2–4). As shown in Tab. 1, 28% of the patients reported to be noncompliant with the driving restrictions within the first two months after implantation. There were no significant differences in socio-demographic, clinical and psychological factors between compliant and noncompliant patients.

**Table 1:** Baseline characteristics of the total sample and stratified for compliance and noncompliance with driving restrictions^a^

	Total sample *N* = 313	Compliance *N* = 226 (72%)	Noncompliance *N* = 88 (28%)	*p*-value
*Socio demographic characteristics*				
Age	64 (55–71)	64 (54–71)	65 (59–70)	0.95
Female	59 (19%)	44 (20%)	15 (17%)	0.61
Having a partner	256 (82%)	186 (83%)	70 (80%)	0.42
Higher education (vocational or higher)	279 (91%)	198 (91%)	81 (92%)	0.35
Employed	112 (36%)	81 (37%)	31 (35%)	0.65
Smoking	33 (11%)	22 (10%)	11 (13%)	0.51
Use of alcohol	205 (66%)	147 (66%)	58 (66%)	0.96
*Clinical characteristics*				
Body mass index	27 (24–30)	26 (24–30)	27 (25–29)	0.96
Left ventricular ejection fraction <35%	29 (23–35)	29 (24–35)	28.5 (22–37)	0.80
Ischaemic heart failure aetiology	135 (48%)	100 (49%)	35 (44%)	0.45
New York Heart Association class:				0.32
– Class I	91 (29%)	60 (27%)	31 (35%)	
– Class II	146 (47%)	106 (48%)	40 (46%)	
– Class III	68 (22%)	51 (23%)	17 (19%)	
– Class IV	4 (1%)	4 (2%)	0 (0%)	
Cardiac resynchronisation therapy	84 (27%)	62 (28%)	22 (26%)	0.73
Primary prophylactic indication	212 (69%)	154 (69%)	58 (67%)	0.78
Reports history of cardiac arrest	68 (22%)	48 (22%)	20 (23%)	0.91
Comorbidities^b^	153 (49%)	110 (49%)	40 (46%)	0.41
*Psychological characteristics*				
Psychotropic medication^c^	27 (9%)	21 (10%)	6 (7%)	0.44
Type D personality^d^	42 (14%)	32 (15%)	10 (12%)	0.47
Anxiety^e^	45 (15%)	31 (14%)	14 (17%)	0.59
Depression^f^	69 (22%)	52 (23%)	17 (19%)	0.46
ICD concerns^g^	7 (2–13)	9 (3–14)	5 (2–11)	0.16
Loneliness^h^	16 (12–22)	17 (12–22)	15.5 (12–22)	0.85

Categorical variables are reported as *N* (%) and continuous variables as median (interquartile range)

*ICD* implantable cardioverter defibrillator

^a^Noncompliance: driven a motor vehicle within the first 2 months after ICD implantation

^b^Comorbidities are scored as present in case a patient reports to suffer from ≥1 of the following conditions: atrial fibrillation, diabetes mellitus and chronic obstructive pulmonary disease

^c^Using psychotropic medication is scored as present in case a patient reports to use ≥1 of the following categories: antidepressants, anxiolytics and hypnotics

^d^Type D personality: score of >10 on negative affectivity and social inhibition subscales of Type D scale (DS14)

^e^Anxiety: score of >10 on Generalised Anxiety Disorder (GAD-7) scale

^f^Depression: score of >10 on Patient Health Questionnaire (PHQ-9)

^g^ICD concerns: total score ICD concerns questionnaire (ICDC)

^h^Loneliness: total score University of California, Los Angeles Loneliness Scale (UCLA-R-S)

As shown in Tab. 2, almost all patients (97%) reported they were informed about the driving restrictions, and generally they felt that information provision was sufficient (81%) and delivered at the right moment (92%). Compliant and noncompliant patients did not significantly differ in their driving behaviour or opinions regarding information provision. However, noncompliant patients were more likely to have considered refusing the ICD because of driving restrictions than compliant patients (19% versus 10%, *p* = 0.02). Also, noncompliant patients showed a tendency towards being less likely to feel they understand the reason behind the driving restrictions (75% versus 84%, *p* = 0.09).

**Table 2:** Driving-related baseline characteristics of the total sample and stratified for compliance and noncompliance with driving restrictions

	Total sample *N* = 313	Compliance *N* = 226	Noncompliance *N* = 88	*p*-value
Owns driver’s license for:				
– Vehicles ≤3,500 kg	304 (99%)	216 (99%)	88 (100%)	0.37
– Vehicles 3,500–7,500 kg	32 (11%)	22 (10%)	10 (11%)	0.74
– Vehicles to transport ≥8 persons	22 (7%)	15 (7%)	7 (8%)	0.74
Partner has driving license	211 (69%)	154 (70%)	57 (66%)	0.79
Main driver in family	237 (79%)	165 (77%)	72 (83%)	0.28
Driving >50% for work	114 (37%)	79 (36%)	35 (40%)	0.56
Driving >50% outside urbanised areas	267 (87%)	191 (88%)	76 (86%)	0.52
Number of kilometres per week	150 (79–300)	150 (73–300)	200 (95–350)	0.57
Informed about driving restriction	296 (96%)	214 (97%)	82 (94%)	0.20
Feels timing of information was good	276 (92%)	198 (91%)	78 (94%)	0.13
Feels information provision was sufficient	247 (81%)	179 (81%)	68 (78%)	0.45
Feels they understand reason	249 (81%)	185 (84%)	64 (75%)	0.09
Feels the restriction is too long	125 (42%)	95 (44%)	30 (35%)	0.13
Considered refusing ICD because of driving restriction	38 (13%)	21 (10%)	17 (19%)	*0.02*

Categorical variables are reported as *N* (%) and continuous variables as median (interquartile range), and significant results are presented in italic

*ICD* implantable cardioverter defibrillator

### Associated factors of compliance with the driving restriction

The results of the multivariable logistic regression analysis are shown in Tab. 3. Only the feeling of understanding the reason behind the driving restrictions was associated with compliance (odds ratio = 2.16, 95% CI 1.02–4.56, *p* = 0.04). This indicates that patients who feel they understand the reason behind the restrictions were more likely to be compliant. Socio-demographic, clinical, psychological and other driving-related factors were not significantly associated with compliance.

**Table 3:** Factors associated with compliance to driving restrictions

	Compliance		
	OR	95% CI	*p*-value
Female	0.81	0.36–1.80	0.60
Higher age	1.00	0.97–1.03	0.88
Having a partner	1.52	0.70–3.28	0.29
Higher education	1.18	0.64–2.20	0.60
Being the main driver	1.14	0.52–2.50	0.75
Having to drive to work	0.96	0.50–1.85	0.91
Feeling of understanding reason behind restrictions	2.16	1.02–4.56	*0.04*
Accepting the driving restrictions	1.15	0.60–2.16	0.68
Higher body mass index	0.98	0.91–1.04	0.46
New York Heart Association class ≥II	1.49	0.71–3.13	0.29
Primary indication	0.94	0.48–1.84	0.85
Cardiac resynchronisation therapy	1.03	0.51–2.09	0.93
Comorbidities^a^	0.87	0.48–1.59	0.65
Type D Personality^b^	1.63	0.64–4.16	0.31
Anxiety^c^	0.74	0.31–1.72	0.48
Depression^d^	1.37	0.62–3.04	0.44
Considered refusing the ICD	0.60	0.26–1.40	0.24

Significant results are presented in italic

*CI* confidence interval, *ICD* implantable cardioverter defibrillator, *OR* odds ratio

^a^Comorbidities are scored as present in case a patient reports to suffer from ≥1 of the following conditions: atrial fibrillation, diabetes mellitus and chronic obstructive pulmonary disease

^b^Type D personality: score of >10 on negative affectivity and social inhibition subscales of Type D scale (DS14)

^c^Anxiety: score of >10 on Generalized Anxiety Disorder (GAD-7) scale

^d^Depression: score of >10 on Patient Health Questionnaire (PHQ-9)

## Discussion

In our sample, 28% reported to be noncompliant with driving restrictions. Patients were able to apply for a special driving license two months after implantation, and the majority received their license 2–4 weeks later. Previously, three quantitative studies, two American and one Irish, examined compliance with physicians’ driving recommendations [7, 8, 17]. The prevalence of noncompliance varied between 58 and 74% in American patients who were recommended not to drive during six months after implantation [7, 8]. Of the Irish patients who were advised to abstain from driving for two months, 23% reported to be noncompliant [17]. This indicates that shorter restrictions are associated with better compliance. Yet, 28% noncompliance is still a significant percentage, especially as this might be an underestimation due to socially desirable answers regarding compliance with driving restrictions [18].

Univariate analysis showed that noncompliant patients more often considered refusing ICD treatment because of the driving restrictions, as well as a trend towards a limited feeling of understanding the reason behind the restrictions. In multivariate analysis, only the feeling of understanding the reason was associated with better compliance and may therefore be key in obeying the restrictions. This was confirmed in a Swedish qualitative study, where patients reported that compliance depended on mutual understanding and agreement between patients and physicians when discussing the driving restrictions. Patients expressed that noncompliance could occur if they felt their beliefs, expectations and preferences were not addressed or when the information was unclear or delivered at an inappropriate moment [5].

Contrary to previous American studies, we found no socio-demographic, clinical, psychological or driving-related factors that were significantly associated with compliance. Craney et al. [7] found correlations between early resumption of driving and the importance of maintaining one’s lifestyle, driving for necessity or social reasons, and being the main driver in the family. On the other hand, Hickey et al. [8] found that noncompliant patients were more likely to be younger, male, college educated, and to have ventricular tachycardia as index arrhythmia, compared to compliant patients. This indicates that patient characteristics may have less impact if the driving restrictions are shorter [7, 8].

After receiving a suitability statement from their cardiologist, Dutch patients can apply for a special driving license. However, if they do not apply for a new driving license they can keep their regular license without violating any laws. In this case, patients may be held liable without any insurance coverage if an accident occurs. Thus, it is important to note that driving is restricted, not prohibited by law. Many patients find this confusing, which may complicate their understanding of the driving restrictions [5]. To improve compliance, it is important that patients feel they understand the reason behind the restrictions, namely that the risks associated with their heart disease (i. e. syncope due to ventricular arrhythmia) can cause harm to themselves and others while driving. When discussing the ICD implantation, physicians may simply ask their patients whether they understand this underlying reason. If not, extra education may positively influence the patient’s compliance. In addition, it would benefit patients’ understanding when European recommendations regarding driving become more uniform and standardised information on this topic is available for every country.

As the Dutch driving restrictions were published in 2000, they were designed with secondary prevention ICD patients in mind. Since MADIT II [19] and SCD-HeFT [20], however, the number of primary prevention ICD implantations has vastly increased. Nowadays, the majority of the ICDs is implanted for primary prevention (e. g., 69% in our sample). These patients are considered to have a lower risk of sudden incapacitation than secondary prevention ICD patients [21], however, a distinction in driving restrictions is currently lacking. This is confusing, as patients eligible for primary ICD implantation are allowed to keep their normal driving licenses without any restrictions if they decide to refuse ICD implantation (provided they are not in NYHA class III or IV). Although indication for ICD implantation was not associated with compliance in this study, clinical practice indicates that primary prevention ICD patients often feel that the driving restrictions are unjust. In the Netherlands, NYHA class III or IV patients are restricted from driving due to severe heart failure symptoms. These patients were not excluded from this study, as their NYHA class could improve (e. g., after CRT-D implantation). We performed a sensitivity analysis with NYHA I and II only, as NYHA III and IV patients might already be used to driving restrictions before ICD implantation. This sensitivity analysis yielded equal results, indicating that including NYHA III and IV patients did not influence our findings.

Evidence supporting the driving restrictions is scarce, which resulted in significant differences between countries, European and non-European, regarding driving restrictions after primary and secondary prevention ICD implantation [2]. Over the past decade, driving restrictions have received little attention in literature, even though patients experience these restrictions as bothersome. Better understanding of patients’ incentives to comply with the driving restrictions after ICD implantation could enhance patient-centred care. This study emphasised the importance to direct attention towards the patient’s understanding of the reason behind the restrictions. Additionally, uniform recommendations, for example in Europe, and a distinction between primary and secondary ICD patients might help enhance patients’ acceptance and understanding of the driving restrictions.
